# Health research policy: a case study of policy change in Aboriginal and Torres Strait Islander health research

**DOI:** 10.1186/1743-8462-6-2

**Published:** 2009-02-26

**Authors:** Sophia Leon de la Barra, Sally Redman, Sandra Eades

**Affiliations:** 1 Sax Institute, PO Box 123, Broadway, NSW 2007, Australia; 2 Baker Heart Research Institute and International Diabetes Institute, PO Box 6492 St Kilda Road Central, Melbourne, VIC 8008, Australia

## Abstract

**Background:**

There is considerable potential for health research to contribute to improved health services, programs, and outcomes; the policies of health research funding agencies are critical to achieving health gains from research. The need for research to better address health disparities in Indigenous people has been widely recognised. This paper: (i) describes the policy changes made by the National Health and Medical Research Council (NHMRC) from 1997 to 2002 to improve funding of Aboriginal health research (ii) examines catalysts for the policy changes (iii) describes the extent to which policy changes were followed by new models of research and (iv) outlines issues for Indigenous health policy in the future.

**Methods:**

This study had two parts: (i) semi-structured interviews were conducted over a four -month period with seven individuals who played a leading role in the policy changes at NHMRC during the period 1997–2002, to describe policy changes and to examine the catalysts for the changes; (ii) a case study was undertaken to evaluate projects by recipients of NHMRC People Support awards and *NHMRC Capacity Building Grants in Population Health Research *to examine the types of research being undertaken five years after the policy changes were implemented. The proposals of these researchers were assessed in terms of whether they reported intending to: evaluate interventions; engage Indigenous community members and organisations; and build research capacity among Indigenous people.

**Results:**

Seven policy changes over a period of five years were identified, including those to: establish an ethical approach to working with Indigenous people; increase the influence of Indigenous people within NHMRC; encourage priority research directed at improving Indigenous health; and recognise Aboriginal and Torres Strait Islander health research as a priority area including a commitment to an expenditure target of 5% of annual funds. Seven catalysts for this change were identified. These included: a perceived lack of effective response to the health needs of Indigenous people; a changed perception of the role of NHMRC in encouraging research to maximise health gains; and leadership within the organisation.

The case study analysis demonstrated that 45% of all People Support recipients intend to engage Indigenous community members and organisations in consultation, 26% included an evaluation of an intervention and two (6.5%) were granted to an individual from an Indigenous background. Six of seven Population Health Capacity Building Grants that were awarded to study Indigenous health between 2004 and 2006 included an intervention component; these grants supported 34 researchers from Indigenous backgrounds.

**Conclusion:**

NHMRC made significant policy changes from 1997 to 2002 to better support Indigenous health as a result of external pressure and internal commitment.

The policy changes have made some progress in supporting better research models particularly in improving engagement with Indigenous communities. However, there remains a need for further reform to optimise research outcomes for Indigenous people from research.

## Background

There is great potential for health research to contribute to better health services, programs, and outcomes. The policies of health research funding agencies can substantially influence the kind of research conducted; there is therefore considerable interest in how the policies of research funding agencies are established, their responsiveness to government and community pressures, and the impact on research practice. For example, the recent review of health research funding in the UK–the Cooksey Report–emphasised the need for an overarching health research strategy and found that "the UK is at risk of failing to reap the full economic, health and social benefits that the UK's public investment in health research should generate" [1].

In Australia, the National Health and Medical Research Council (NHMRC) is the major funder of health research. A review of health and medical research in Australia commissioned by the government in 1998 (the Health and Medical Research Strategic Review, or Wills Committee Review) recommended changes in policy to better focus the research effort on outcomes such as health and wealth creation [2]. Subsequent changes to NHMRC policy contributed to a substantial increase in the number of patents arising from funded research and therefore potential wealth creation. However, less progress has been made in encouraging research to inform health policy and practice and produce health gains [3].

Of all the sub-groups in the Australian population who require a strengthened research effort to produce improved health outcomes, the need is clearest for Indigenous Australians. Research directed at improving the health of Indigenous people is recognised as a major priority in Australia. The life expectancy for Indigenous Australian men is 19 years less than for non-Indigenous men and 18 years less for Indigenous women than their non-Indigenous counterparts [4]. There has been little change in the mortality differential in recent years, in contrast to the progress made in comparable countries such as New Zealand and Canada, where Indigenous health has improved relative to that of the rest of the population [5].

Historically, the research effort in Indigenous health in Australia has been less than optimal [6,7]. Aboriginal populations in Australia have been the subject of research since the 19th Century; however, this research was primarily anthropological and focused on accumulating information before Aboriginal people were 'lost to science' rather than on how best to address Indigenous health problems [8]. Aboriginal Australians report feeling that they have been exploited by disrespectful experimentation–subjected to invasive examinations and procedures, objectified, scrutinised, and inaccurately represented–without this research conferring any health benefits to Aboriginal populations [7,9,10]. In the 1970s a new dialogue began, led by Indigenous people and focused on issues of control, from community consultation and consent, to intellectual ownership and application of research findings [11-14]. In the 1980s, these debates culminated in the articulation of ethical guidelines for research in Indigenous populations. Two themes emerged from these debates: for Aboriginal communities to have ultimate ownership and control of research, a concerted effort to train Indigenous researchers will be required and to enable "useful research" to be conducted, Indigenous communities need to help identify and define research questions [6,11,12,14].

Informed by these discussions, there has been a growing consensus over the past ten years both within Australia and internationally that research is more likely to have a long-term impact in improving the health of Indigenous people if it evaluates the impact of health programs rather than simply describing health problems, involves Indigenous researchers in all stages of the research, and builds capacity among Indigenous researchers [15,16]. Research funding policies designed to stimulate research of this kind could greatly assist in creating useful evidence to improve Indigenous health.

It is clear that research funding policies over the past twenty years could have been better targeted to improve Indigenous health. Too often, research has simply described health problems without seeking to find solutions; for example, a review of published research in Indigenous health from 2001–2003 found that a mere 13% of Australian peer-reviewed papers evaluated the impact of interventions, with the remainder primarily describing health problems or their causes [17]. Further, it is widely recognised that in Australia, research has frequently failed to engage Indigenous people as equal partners in research or offered an opportunity for involvement in all stages of data collection, including planning, implementing, analysing and disseminating [6,7].

Beginning in 1997, NHMRC responded to the challenge of improving Aboriginal health with a number of substantial policy changes designed to increase funding for Indigenous research and to better target the research effort. NHMRC's response is of considerable interest in understanding the factors that can contribute to policy change in research funding. Like many research funding agencies internationally, NHMRC had historically almost exclusively funded investigator-initiated research with little capacity to strategically target funding to specific areas; its response to the challenge posed by the need to improve Indigenous health was therefore unique in its history.

This paper (i) describes policy changes NHMRC has made to improve funding of Aboriginal health research (ii) examines the catalysts for policy changes (iii) describes the extent to which policy changes were followed by new models of research and (iv) outlines issues for Indigenous health policy in the future. The paper focuses on an analysis of funding for scholarships, fellowships and other awards to pay the salaries of individual researchers (referred to by NHMRC as People Support). We decided to examine People Support because it provided an opportunity to explore the impact of the policy changes on the development of workforce capacity in Aboriginal health research and to examine support for researchers from Indigenous backgrounds. The impact of policy change on the amount of funding through People Support for Indigenous health research is described elsewhere [18].

## Methods

### Key informant interviews

In order to describe the policy changes and to examine the catalysts for the changes, semi-structured interviews were conducted over a four-month period with seven individuals who played a leading role in the policy changes at NHMRC during the period 1997–2002. These seven individuals were involved in the policy changes that were led by the Aboriginal and Torres Strait Islander Research Agenda Working Group (RAWG); in the course of its business, RAWG interacted with other NHMRC principal committees. Accordingly, interviews were undertaken with the former Chair of the Research Committee, as well as the Chair and other leading Indigenous and non-Indigenous members from RAWG during this period.

The interviews were conducted by one of the authors (SLB) and addressed the policy changes that had occurred; the key factors driving policy change (key evidence, individuals, and circumstances); climate and timing for policy changes; barriers to changing policy; as well as approaches and strategies used to encourage policy change. The interviews were digitally recorded and transcribed. Both the transcribed interviews and notes taken during the interviews were used to conduct the analysis.

The interviews were content-analysed according to major thematic areas and trends in current policy-making literature.

### Case Study Analysis

We conducted a case study evaluating the projects undertaken by NHMRC People Support recipients of the following funding vehicles: Scholarships (for postgraduate study, most usually leading to a PhD), Training Awards (for postdoctoral researchers), Career Development Awards (for researchers two to twelve years after the award of a PhD), and Career Awards (for senior researchers). We also examined recipients of *NHMRC Capacity Building Grants in Population Health Research*; this new funding model was introduced in 2002 as a short-term initiative. The grants support junior researchers to work for five years within a mentoring environment with senior researchers.

A keyword search of the NHMRC Research Management Information System (RMIS) was used to identify Indigenous health researchers who received NHMRC People Support awards with the following terms in either the title, lay summary, keywords, or fields of research: Aborigines or Aboriginal; Torres Strait Islander; Indigenous; Koori. For Capacity Building Grants in Population Health Research, a standardised question on the application form (section 1.3) was used to identify "research involving Aboriginal and Torres Strait Islander Peoples." Applications that ticked 'yes' were included.

For the purposes of this case study, only People Support Awards awarded in 2005 or 2006 and Capacity Building Grants in Population Health Research awarded in 2004, 2005 and 2006 were examined; this enabled an examination of successful applicants at a time when the policy impact was likely to be greatest.

The authors of this paper were granted access to original application forms for review, and double-coded all applications for 14 items including information about thematic areas of the *Road Map*, project design, and research practices. Operational definitions for coding the data were developed and reviewed by all the authors. Each case was coded independently by two people against a detailed operational definition; in case of disagreement, the application was jointly reviewed for consideration until the coders could agree on how the item should be coded.

The grants were assessed according to whether they:

• **Evaluated interventions: **that is, the applications included evaluations or trials of interventions, services or programs designed to improve the health of Aboriginal people.

• **Engaged Indigenous community members and organisations: **that is, the application (a) described an advisory group with Indigenous membership in project design; and (b) completed a special section of their application to NHMRC outlining their commitment to engage Indigenous community members and organisations in research partnership. Completion of this section is considered mandatory by NHMRC for all health and medical research with Indigenous Australians.

• **Built research capacity among Indigenous people: **that is, the application (a) proposed to employ or train an Indigenous person as part of the research team; or (b) was a People Support Award to an individual who self-identified as Indigenous.

### Classification of Indigenous status

NHMRC People Support applicants were classified as Indigenous if they self-identified as an Aboriginal or Torres Strait Islander person on the application form.

## Results

### (i) Policy changes to improve funding of Aboriginal health research

**Figure 1:**
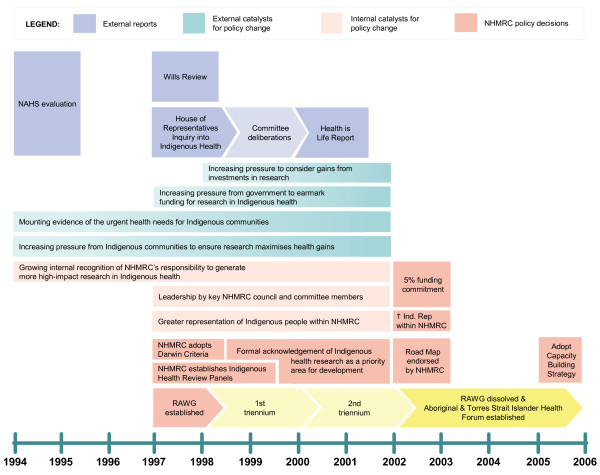
**Policy timeline for Indigenous health research**.

Respondents nominated seven changes to NHMRC policy over a five-year period and spanning two triennia of the operation of RAWG; the evolution and sequence of these policy decisions is illustrated in Figure 1.

In the early part of this period, three changes occurred:

• ***Establishment of the Aboriginal and Torres Strait Islander Research Agenda Working Group (RAWG): ***RAWG was established in 1997 to guide the development of a strategic research agenda for NHMRC and to encourage better practices in Indigenous health and research. RAWG was established as a subcommittee of the former Strategic Research Development Committee (SRDC) over the course of two NHMRC triennia from 1997–1999 and 2000–2002.

• ***Adoption of the Darwin Criteria***: Most participants nominated the adoption of the Darwin Criteria in 1997–an initiative of RAWG in its first triennium–as a key policy change. The Darwin Criteria were developed as a set of principles to guide research with Indigenous communities. They were intended to be used to assess project grant applications in terms of their level of engagement and capacity building with Indigenous communities, the significance and benefit of research proposals to Indigenous health and the transferability of the methods to other settings. NHMRC adopted the Darwin Criteria as part of the assessment process to gauge applicants' approach to working ethically and in partnerships with Indigenous communities.

• ***Establishment of the Indigenous Health Review Panel: ***This panel was established in 1997 as part of the assessment process for project proposals in Indigenous health; it was an initiative of RAWG in its first triennium. The Indigenous Health Review Panel utilised the Darwin Criteria and the expertise of Indigenous panel members to provide advice on cultural appropriateness of applications and methods and to comment on approaches to community consultation. The Indigenous Health Review panel was also able to comment on the scientific quality of the applications and to stipulate conditions upon which funding is contingent. The reports of the Indigenous Health Review Panel were used by Grant Review Panels in making further assessment of project grant applications and final funding recommendations.

At the 144^th ^Council session of NHMRC held in October 2002, major policy issues addressed by RAWG in its second triennium of activity (2000–2002) were considered. An options paper developed by RAWG was tabled outlining policy options for Aboriginal health research, including consideration of the RAWG Road Map, mechanisms to increase representation of Indigenous people in the NHMRC and consideration of efforts to increase the level of specific research funding to Indigenous health [19]. Interviewees reported that policy documents prepared by RAWG were received with "a high degree of vocal support" on Council. The following policy decisions were endorsed at that time:

• ***Endorsement of the NHMRC Road Map: A Strategic Framework for Improving Aboriginal and Torres Strait Islander Health Through Research: ***In its second triennium, RAWG conducted a national consultation process to identify priorities for research in Indigenous health. The RAWG Road Map for research was developed through this consultation process, involving a series of four national workshops and written submissions to set out a strategic approach to Indigenous health research for NHMRC. Indigenous community members, researchers and policy-makers contributed to the consultation process. All participants acknowledged the comprehensive consultation process used to develop the Road Map, and identified the Road Map as a compelling policy document to guide future NHMRC investments.

• ***Acknowledgement of Aboriginal and Torres Strait Islander health research as a priority area for development: ***Several participants highlighted the importance of the acknowledgement by the Council of Indigenous research as a priority in improving expenditure and Indigenous representation within the agency. One respondent reported that this acknowledgement enabled Council to allow funds to be earmarked for Indigenous health research.

• ***Commitment to target of 5% annual expenditure: ***At the October 2002 Council meeting, it was agreed that NHMRC would work towards a target of expending at least 5% of its annual budget on Indigenous health research. All participants acknowledged this funding commitment as a landmark decision by NHMRC.

• ***Increase Aboriginal and Torres Strait Islander representation across all NHMRC Principal Committees and Council: ***In accordance with the principle to engage Indigenous community members in all stages of research, all participants emphasised the importance of Indigenous participation in decision-making within NHMRC and noted increased Indigenous participation throughout the 1997–2002 period.

### (ii) Catalysts for policy change

Participants identified both external and internal factors as being influential in bringing about the policy change. Four **external **factors were identified by all participants:

• ***Mounting evidence of the lack of effective response to the urgent health needs of Aboriginal and Torres Strait Islander communities: ***Participants identified the 1994 evaluation of the National Aboriginal Health Strategy (NAHS) as influential in shaping the policy climate. This evaluation indicated that the strategy had little impact in improving the state of health for Aboriginal people, and had not been adequately implemented. One informant said that, upon scrutinising the national investment approach to Aboriginal health issues, it became apparent that "there is no evidence of where that money's been spent".

• ***Increasing pressure from government to earmark funding for research in Aboriginal and Torres Strait Islander health: ***Participants nominated the House of Representatives Standing Committee on Family and Community Affairs inquiry into Indigenous health commencing in 1997 as a key catalyst. Written submissions and committee deliberations culminated in the *Health is Life *report in 2000 [20], which issued a series of 35 recommendations to improve Indigenous health. The final recommendation of the report was that "the National Health and Medical Research Council allocate at least five per cent of total annual research funding for Indigenous health research" [20]. NHMRC was formally invited to consider and respond to recommendation 35 in the *Health is Life *report.

• ***Increasing pressure to consider gains from investments in research: ***Participants nominated the 1998 Health and Medical Research Strategic Review [2] as a key catalyst for policy change at NHMRC. As one participant explained, the review stemmed from "an underlying principle that we don't just need to do research for the interest of research... we should really try to address some of the gaps in our understanding or knowledge that may actually lead to improved outcomes and to better ways to deliver resources". The 1998 Health and Medical Research Strategic Review emphasised both the importance of evidence-based health care, and the role of efficient grant allocation mechanisms in managing public investments.

• ***Increasing pressure from Indigenous communities to ensure real health gains from any research: ***Participants cited a growing body of opinion that Indigenous communities had derived little benefit from research. As one participant put it, "we've been researched to death". Participants reported that Aboriginal community members, academics and organisations emphasised the need for a new approach to research; to ensure real health gains, community engagement in all stages of the research process was required. As one senior informant explained, "they [non-Indigenous researchers] keep researching Blacks, but there's still Blacks sitting there with the same illnesses as when I was a kid! Building Aboriginal research capacity is the way forward...".

All interviewees identified the following three **internal **factors as influential:

• ***Growing internal recognition of NHMRC's responsibility to contribute meaningful research of Aboriginal and Torres Strait Islander health issues: ***Participants felt that external evaluations of Indigenous health research investment, particularly the 1998 Health and Medical Research Strategic Review, created a "desire to be seen to do something" within NHMRC. This Review was particularly influential in prioritising the poor state of Aboriginal health as an area requiring increased investment by NHMRC. Interviewees described an increasing acknowledgement across NHMRC that high quality research supported by efficient funding mechanisms could contribute to improving health outcomes for Indigenous Australians.

• ***Greater representation of Indigenous people within NHMRC: ***All interviewees emphasised the key role played by Indigenous members of Council and Principal Committees in driving the policy changes. Initially, this was through RAWG; however, in 2002, Council decided to increase Indigenous membership on Council and its Principal Committees. In the first triennium of its existence (1997–1999), the RAWG had a non-Indigenous Chair and in its second triennium (2000–2002) RAWG was chaired by an Indigenous member of the Council of NHMRC, Mr John Delaney. During this period, RAWG had a majority Indigenous composition; there was also membership on RAWG from other Commonwealth Government and community controlled agencies. RAWG membership included researchers and experts in Indigenous health, representatives from the Office of Aboriginal and Torres Strait Islander Health, the federal government's Standing Committee for Aboriginal and Torres Strait Islander Health and the National Aboriginal Community Controlled Health Organisation. Participants noted the importance of Indigenous people constituting a majority within RAWG during its second triennium, from 2000 onwards.

• ***Leadership by key NHMRC Council and principal committee members: ***Participants acknowledged the key role played by chairs of the Research Committee and Strategic Research & Development Committee in prioritising Aboriginal health within NHMRC. Interviewees said that as external pressure to address Indigenous health issues mounted, key committee members across the agency encouraged NHMRC to be responsive, and develop an agency-wide strategy to improve Indigenous health.

Two other issues of importance were raised by participants:

• ***Legislation: ***Several participants commented on the role of the NHMRC Act and differing interpretations over time. In early discussions, there was a view in the organisation that the legislation precluded the allocation of funding for specific purposes, such as 5% for Indigenous research. This view was revised in internal discussions during the 2000–2002 triennium.

• ***Implementation***: All participants emphasised the importance of revisiting existing policies to evaluate the implementation. Respondents noted that the Road Map had not included measurable indices for implementation and that it would therefore be difficult to assess the extent to which NHMRC had implemented the recommendations. Further, several participants also noted that a strategy for building capacity in Indigenous health [21] had been agreed by NHMRC at its Council meeting in December 2005 but had not been implemented.

### (iii) Impact of policy change on models of research

The keyword search for 2005 and 2006 identified 38 People Support recipients who research Indigenous health. However, upon closer review of these applications, seven were excluded because no component of the outlined research project mentioned or included the study of an Indigenous population.

In 2005 and 2006, 31 awards made through the People Support scheme to researchers studying Indigenous health (16 Scholarships, 9 Post-doc Training Awards, 3 Career Development Awards, and 3 Career Awards). Two NHMRC Scholarships were awarded to applicants who self-identified as Indigenous.

While NHMRC Capacity Building Grants in Population Health Research are *Strategic Awards*, and not part of the traditional *People Support *scheme, they do encompass a framework for the educational and financial support of early career health researchers in training. In the period 2004 – 2006, seven Capacity Building Grants in Population Health Research were awarded to support Indigenous health research; a total of 58 team or junior investigators were allocated funding through these grants (24 self-identify as non-Indigenous and 34 self-identify as Indigenous).

The grants were classified as follows:

**a) Evaluated interventions: **eight of 31 (26%) of People Support recipients in Aboriginal health research included an intervention component in their research program; six of seven successful Capacity Building Grants in Population Health Research for Indigenous health included the development or implementation of a health intervention in research design.

**b) Engaged Indigenous community members and organisations: **14 (45%) People Support recipients in Aboriginal health research described a project advisory group with Indigenous membership. NHMRC Scholarship holders were the most likely to include an Indigenous project advisory group in their proposed research program (10 [63%] scholarship holders compared to four [26%] of Training Award, Career Development Award, and Career Award recipients). Six of seven Capacity Building Grants in Population Health Research for Indigenous health, described the inclusion of an Indigenous project advisory group in project design.

Twenty (65%) People Support recipients addressed the Darwin Criteria in their application forms. Completion rates were much higher among Scholarship (94%) and Training Award recipients (56%); no recipients of Career Development Awards or Career Awards completed this section of their application. Just over half of successful Capacity Building Grant applications completed this section.

**c) Built research capacity among Indigenous people: **17 (55%) of People Support recipients reported training or employing an Indigenous person not directly supported by the NHMRC as part of the research team.

Among awards for Indigenous health research commencing in 2005 and 2006, only two People Support recipients self-identified as Indigenous. In contrast, 34 team investigators studying Indigenous health and supported by Capacity Building Grants in Population Health between 2004 and 2006, self-identified as Indigenous.

## Discussion

This paper outlines the substantial policy changes made by NHMRC to improve funding of Aboriginal health research during the period 1997–2002. Policy shifts do not occur evenly over time; the period 1997–2002 represents a period of rapid acceleration in policy change in relation to Aboriginal health research at NHMRC. During this period the establishment of the Aboriginal and Torres Strait Islander Research Agenda Working Group (RAWG) was the first step in a series of initiatives that included the agreement of an ethical approach to working with Indigenous people, through the adoption of the Darwin Criteria. The NHMRC established policies to increase the influence of Indigenous people within NHMRC by increasing the Indigenous representation on RAWG and through the establishment of Indigenous Health Review Panel. A framework to encourage priority research directed at improving Indigenous health was established through the endorsement of the *NHMRC Road Map: A Strategic Framework for Improving Aboriginal and Torres Strait Islander Health through Research*. However, probably of most importance, the policy changes resulted in an explicit acknowledgement of Indigenous health research as a priority area for development and a commitment to a 5% expenditure target of annual funds [19].

Taken together, this represents a substantial set of policy changes to address an urgent health need through research–the magnitude of the policy response is unique in the NHMRC's history. Historically, the vast majority of research funded by NHMRC has been investigator-initiated research selected for funding primarily on the basis of scientific excellence; as noted by the Wills Review in 1998, relatively little funding prior to 1998 was directed towards strategic research focused on government or community priorities. The lack of response to Indigenous health prior to this time was therefore part of a general philosophy about research funding and indeed hampered the NHMRC's capacity to act strategically in other areas of health need as well [22]. The funding philosophy at the time is illustrated by the view inside NHMRC that its legislation may preclude allocation of funds to a specific area (e.g. 5% to Indigenous health). There was also a view during the period of reform addressed in this paper that a designated allocation of funds to Aboriginal health would necessarily involve a decline in scientific standards.

It is therefore of some interest to understand the factors that led to these policy changes. Kingdon's *multiple streams model *of policy-making outlines three streams that contribute to whether a policy change is adopted: the *problem *stream (a given situation has to be identified and explicitly formulated as a problem or issue); the *policy *stream (an explicit formulation of policy alternatives and proposals must be available); and the *political *stream (a political event or climate that affects the balance of costs and benefits) [23]. Based on interviews with key participants, three broad sets of factors corresponding to Kingdon's streams were identified. First, there was a clear identification of the problem–a lack of effective response to the urgent health needs of Aboriginal and Torres Strait Islander communities both from governments and from Indigenous communities. The data about disparities in health status were compelling enough for government to recognise that action was required.

Second, a clear action was identified for NHMRC–namely to increase the proportion of funding provided for Aboriginal health research to 5%. The identification of a simple technically feasible response by the House of Representatives, followed by a letter from the Minister, was of fundamental importance to the adoption of the policy change [20,24,25]. The RAWG Road Map also made specific recommendations that could be readily adopted by NHMRC [16].

Third, there was a change in the climate within NHMRC to one that was more receptive to policy change in Aboriginal health research. Recommendations of the House of Representatives and the emphasis in the 1998 Health and Medical Research Strategic Review on demonstrating health gains from investment in research were both important in changing the political climate within NHMRC. People interviewed emphasised the key leadership role played by a number of Council and principal committee members within the organisation in building this receptive climate, particularly those from an Indigenous background. Respondents identified the Research Agenda Working Group (RAWG) as being the single most important driver in policy change.

It was not possible to determine the extent to which the policy changes caused observed improvements in funding for Indigenous research through NHMRC. Although participants in the interviews believed that the policy changes had been very important in changing the climate within the organisation and externally, there is no doubt that other forces in the wider community may have impacted on research approaches. The 1990s was a period when many important health, social and legal policies in relation to Indigenous people were overhauled. For example, from 1985, the training of Indigenous doctors had been taken up as an innovative policy for improvement of Indigenous health by a small number of medical schools. From 1990 onwards the first of a growing number of medical graduates began to take up influential roles in health service and policy development. In 1998, the High Court of Australia ruled in favour of Mabo.

Nonetheless, it is clear that the policy changes have contributed to an improvement in research in Aboriginal health funded by NHMRC through People Support Awards. The case study in this paper demonstrates that five years after the policy changes, around half of all recipients of People Support Awards to study Indigenous health demonstrated their intention to engage Indigenous community members and organisations–either in consultation (through Indigenous project advisory groups) or as partners in research (by employing or training Indigenous people as part of the research team). Five years after the policy changes, one-quarter (26%) of projects included the development or implementation of an intervention and its evaluation. Six of seven Capacity Building Grants in Population Health that were awarded to conduct Indigenous health research during the study period also included an intervention component in the proposed research program. Other research has demonstrated that there has also been an increase in the amount of funding through People Support Awards to Indigenous health. The proportion of funds for Indigenous health research through the People Support awards roughly doubled between 2002 and 2006 [18]. Although it is possible that applicants did not implement the research in the way proposed in the funding application, there does appear to be a clear intent to develop capacity among Indigenous researchers and to work more closely with Indigenous communities. Further research including interviews with researchers about the implementation of the research, including the consultation processes, would be of considerable interest.

However, there remains a considerable way to go before there is an optimal approach to funding Indigenous health research. Half of the successful applications to People Support Awards did not include strategies to engage Aboriginal communities and relatively few People Support Awards are granted to people from Indigenous backgrounds. Most research still focused on describing the health problems or their causes rather than on testing interventions. The case study analysis does not paint a picture of a research sector working closely with Indigenous communities to find solutions for pressing health issues. In this regard, the Capacity Building Grants in Population Health Research appear to offer an interesting model, since they supported more individuals from Indigenous backgrounds, demonstrated greater evidence of close working relationships between communities and researchers, and fostered an apparently greater focus on intervention research.

It will be important that NHMRC maintains and indeed increases its efforts to support Indigenous health research; based on these findings, several strategies might be investigated.

First, it may be of value to consider the development of Indigenous-specific funding schemes in Australia. Almost exclusively, funding policy has sought to encourage greater number of applications and better success rates in its traditional funding schemes. However, the relative effectiveness of the Capacity Building Grants in Population Health illustrates that different models of funding may be more effective for Indigenous health research. There are excellent models internationally that could inform the development of Indigenous-specific funding schemes. For example, in Canada, one of the institutes of health focuses on Indigenous health, and an innovative training model similar to a travelling university has been developed by the Centre for Aboriginal Health Research to teach applied research skills to Indigenous people in their communities [15,26]. Internationally, health research funding agencies have made substantial investments in infrastructure to build and develop research centres for Indigenous health equipped to work in partnership with communities, and capable of building the skill base and pool of researchers. Further consideration is needed to determine if similar training strategies and funding mechanisms can be suitably adapted to the Australian context.

Second, there will be a need for continued improvement of existing policies through review and monitoring of their impact. With the adoption of the 2002 policy decisions, the NHMRC instated the Aboriginal and Torres Strait Islander Health Forum to provide advice about the implementation, evaluation, and reformulation of the Road Map as a living document. As the second Forum is now in session, the current membership has the responsibility to provide advice about an action plan for implementation and evaluation of current agency policies and has instigated an important review of the Road Map. However, it is evident that much could be done to more fully implement existing policies; for example, it has been mandatory since 1997 to complete what are known as the Darwin Criteria in applying for funding for Indigenous health research. These criteria require applicants to describe how their research will focus on priorities for Aboriginal health, build capacity and engage with Aboriginal communities. However, our analysis suggested that 35% of funded applications addressing Indigenous health through the People Support scheme did not complete this section of the form. Similarly, in 2005, the Forum produced the *Strategy for Building Capacity in Aboriginal and Torres Strait Islander Health Research*–a document outlining the objectives of a capacity building program [21]; these recommendations are yet to be implemented. More could be done to ensure that the selection and assessment criteria throughout NHMRC schemes support the Darwin Criteria. It would also be of value to consider strategies to assist researchers and Indigenous communities to work together; for example, by the provision of funding for liaison positions, promotion of tools for collaboration such as standardised Memoranda of Understanding or support for research training of staff in Aboriginal Medical Services.

Third, this research has highlighted the need for new initiatives to build capacity among researchers from Indigenous backgrounds. Relatively few of the People Support Awards were to researchers from Indigenous backgrounds and this has been confirmed by other analyses over a longer time frame [18]. What strategies should the NHMRC implement to increase the numbers of Aboriginal and Torres Strait Islander students in research? Evidence suggests that funding models centred around collaborative research environments with blended teams of skilled and early career professionals are more likely to work successfully on meaningful, long-term research projects and train highly skilled researchers in the process. The NHMRC Capacity Building Grants in Population Health appear to be an effective means of attracting multidisciplinary research teams to work together on highly beneficial, applied research. Some of these grants supported additional early career researchers in groups with already established strengths in Indigenous health research while others were used to establish capacity in groups interested in building new Indigenous health research teams. The reasons for the relative success of Capacity Building Grants in Population Health in supporting researchers from Indigenous backgrounds are not clear; however, it seems likely that these grants may be perceived as offering better opportunities for researchers from Indigenous backgrounds. The grants are longer (five years), are from larger teams with established infrastructure (including members of Indigenous communities and organisations), and provide greater financial support to team investigators than might be received through a Scholarship. They also offer the opportunity to work collaboratively with other early career researchers from Indigenous backgrounds.

The NHMRC might consider drawing on international models for building capacity among researchers from Indigenous backgrounds. For example, the Canadian Institutes for Health Research demonstrated a commitment to "building research capacity and infrastructure in Aboriginal health research" by establishing the Institute for Aboriginal Peoples Health to administer eight Aboriginal Capacity and Developmental Research Environments (ACADRE) centres. Each of these centres provides an array of scholarship and training opportunities to undergraduate and graduate students (the majority of which self-identify as Aboriginal), as well as to community members and organisations interested in conducting health research [15,26]. Another example is provided by the New Zealand Centre of Research Excellence; in 2002, a five-year target was set to graduate a total of 500 Maori PhD scholars across all academic disciplines. In addition to active recruiting and extensive student support services, all students were provided with a mentor to guide their academic development and provide social support throughout their PhD program. The initiative has been highly successful, and New Zealand is on track to establish a critical mass of Maori scholars in disciplines including health, history, social sciences, and education [27]. As part of the current review of the Road Map, NHMRC might consider the inclusion of similar strategies.

In conclusion, it is evident that with sufficient external pressure and internal commitment, it is possible to make substantial changes to health research funding policy. The NHMRC made significant changes to its policy in 2002 to better support Indigenous health. By 2007, funding for Indigenous health research–at least through its People Support Awards–appears to be moving towards a better model of practice. However, there remains a considerable way to go before Australia could be said to have in place strategies that optimised the research effort in improving the health of Indigenous people. Government, the community and researchers should continue to advocate for improved funding and for the development of new models reflecting international best practice.

## Competing interests

The authors declare that they have no competing interests. Sandra Eades was a member of RAWG and co-authored the Road Map.

## Authors' contributions

SLB contributed to collection, analysis, interpretation, and presentation of data. SR and SE contributed to the intellectual development and design of this research project, and provided substantial revisions and editing in drafting this paper. Any opinions expressed in this paper are the sole responsibility of the authors.
